# Evaluating Salivary Cortisol and Alpha-Amylase as Candidate Biomarkers in Anorexia Nervosa: A Systematic Review and Meta-Analysis

**DOI:** 10.3390/ejihpe15120260

**Published:** 2025-12-17

**Authors:** Takahiro Seura, Yuuna Nanba

**Affiliations:** Department of Home Economics and Technology, Hokkaido University of Education, Asahikawa Campus, 9 Hokumon-cho, Asahikawa 070-8621, Hokkaido, Japan

**Keywords:** saliva, alpha-amylase, cortisol, biomarker, anorexia nervosa, eating disorders, early identification, meta-analysis

## Abstract

**Objectives**: This systematic review and meta-analysis investigated whether salivary cortisol and alpha-amylase levels differ between patients with anorexia nervosa (AN) and healthy controls. **Methods**: A systematic literature search was conducted in PubMed, ScienceDirect, SpringerLink, and Scopus for relevant studies published up to December 2024. Standardized mean differences (SMDs) and 95% confidence intervals (CIs) were calculated using a random-effects model. Statistical heterogeneity was assessed using Cochran’s *Q* and the *I*^2^ statistic. **Results**: Data on salivary alpha-amylase were extracted from six studies including 218 patients with AN and 220 healthy controls. No significant group difference was observed (SMD = −0.48; 95% CI, −1.05 to 0.10; *I*^2^ = 86%), though sensitivity analysis indicated significantly lower levels in AN (SMD = −1.12; 95% CI, −2.16 to −0.09; *I*^2^ = 82%). Salivary cortisol was assessed in 24 reports from 17 studies (517 patients with AN, 672 controls), revealing significantly higher levels in AN (SMD = 0.69; 95% CI, 0.54–0.85; *I*^2^ = 30%). Sensitivity analyses showed stable effect sizes. Meta-regression indicated that neither age nor body mass index significantly moderated the results. **Conclusions**: Salivary cortisol may serve as a reliable, noninvasive biomarker for AN, with potential utility in early identification and prevention of disease progression.

## 1. Introduction

Human saliva primarily consists of water (approximately 99%), along with proteins and electrolytes (de Almeida et al., 2008). Besides its roles in mastication and digestion, saliva is a biological fluid that is essential for maintaining oral health, owing to its antimicrobial properties, buffering capacity, and contribution to oral mucosal repair (Lingström & Moynihan, 2003). Moreover, saliva contains various biomarkers of immune, endocrine, and metabolic functions. Salivary cortisol and alpha-amylase, in particular, are frequently used in clinical and psychological research because they are considered indicators of the host’s stress response and the severity of psychological disorders (Chojnowska et al., 2021). Furthermore, compared to blood or urine samples, saliva collection is noninvasive, stress-free, and cost-effective, which makes it suitable for the assessment of populations, such as adolescents or individuals with eating disorders.

The stress response is regulated by two primary systems: the hypothalamic–pituitary–adrenal (HPA) axis and the sympatho-adrenomedullary (SAM) system. Salivary cortisol is a primary marker of HPA-axis activity that is frequently used to evaluate chronic stress levels (Dedovic et al., 2009). Elevated salivary cortisol levels are associated with various mental health conditions, including depression and anxiety (Hinkelmann et al., 2009; Knorr et al., 2010; Vreeburg et al., 2010). In contrast, salivary alpha-amylase constitutes a biomarker of SAM system activity that reflects acute stress responses (Rohleder et al., 2004). Under stressful conditions, salivary cortisol and alpha-amylase operate independently, with cortisol exhibiting a slower response and alpha-amylase responding more rapidly (Takai et al., 2004; Engert et al., 2011).

Anorexia nervosa (AN) is a severe psychiatric disorder that is characterized by restrictive eating behaviors, an intense fear of weight gain, and a distorted body image. A recent systematic review reported that the average lifetime prevalence of AN was 8.4% and 2.2% in women and men, respectively, with a higher prevalence among adolescent females (Galmiche et al., 2019). AN is associated with substantial morbidity, including cardiovascular issues (Casiero & Frishman, 2006; Gibson et al., 2019), gastrointestinal problems (Norris et al., 2016), and pregnancy-related complications (Pan et al., 2022). Several studies have demonstrated that eating disorders are linked to elevated mortality rates, with AN exhibiting the highest mortality risk among all eating disorders (Birmingham et al., 2005; Arcelus et al., 2011).

Low body weight or starvation resulting from AN alters autonomic nervous system functioning, primarily through activation of the HPA axis, leading to chronically elevated cortisol levels (Lawson et al., 2013; Schorr & Miller, 2017). This hypercortisolemic state has been associated with appetite suppression and increased anxiety, both of which may contribute to the maintenance of AN (Hasan & Hasan, 2011). In patients with AN, circadian rhythms of appetite-related hormones, such as ghrelin, obestatin, neuropeptide Y, and α-melanocyte-stimulating hormone, are also disrupted, and these alterations have been linked to disordered eating behaviors and sleep disturbances (Germain et al., 2010; Galusca et al., 2015). Abnormalities in the SAM system activity, as reflected by altered salivary amylase secretion, may also contribute to circadian rhythm disruption (P. Monteleone et al., 2011b; Paszynska et al., 2016). From a biopsychosocial perspective, the development of AN is strongly influenced by genetic predisposition, personality traits, and sociocultural contexts that promote thinness (Culbert et al., 2015). Thus, assessing salivary cortisol and alpha-amylase levels may provide valuable insights into the biological and psychosocial mechanisms underlying eating disorders.

Although numerous studies have investigated the association between salivary cortisol and alpha-amylase levels and symptoms of AN, there remains no clear consensus regarding the magnitude or direction of these changes. Furthermore, many of these studies were based on small clinical samples, which limits the generalizability of their findings across broader populations. To date, the only meta-analysis to explore the relationship between cortisol and AN was conducted by Thavaraputta et al. in 2023 (Thavaraputta et al., 2023). Their analysis found significant associations between AN and both urinary and serum cortisol levels; however, salivary cortisol levels were not evaluated. To our knowledge, no meta-analysis has investigated the association between salivary alpha-amylase levels and AN yet. A consistent relationship between salivary biomarkers and eating disorder pathology could provide valuable, noninvasive diagnostic information. Furthermore, a better understanding of salivary biomarkers in AN could facilitate the development of novel screening tools or early intervention strategies.

Therefore, this systematic review and meta-analysis aimed to determine whether salivary cortisol and alpha-amylase levels differ between individuals with AN and healthy controls.

## 2. Materials and Methods

### 2.1. Search Strategy

A systematic literature search was conducted across four electronic databases: PubMed, ScienceDirect, SpringerLink, and Scopus. The search aimed to identify relevant studies published between 1999 and December 2024. The search strategy incorporated Medical Subject Headings (MeSH) and relevant keywords related to eating disorders, salivary biomarkers, cortisol, and amylase. Specifically, the search terms included “eating disorders,” “anorexia nervosa,” “binge-eating disorder,” “saliva,” “biomarkers,” “cortisol,” and “amylase.” Boolean operators (AND, OR) were used to combine terms and refine the search results. The complete details of the search strategy, including search terms and applied filters, are provided in Supplementary Table S1. This systematic review and meta-analysis was performed in accordance with the Preferred Reporting Items for Systematic Reviews and Meta-Analyses (PRISMA) statement (Page et al., 2021) (see Supplementary Table S2 for PRISMA Checklist). This study was registered with the UMIN Clinical Trials Registry (registration number UMIN000057920).

### 2.2. Study Selection

Studies were included if they met the following criteria:

(i) Population: individuals of any age or sex, including both adults and children;
(ii) Exposure: patients diagnosed with AN based on clinical evaluation and/or according to the diagnostic criteria outlined in the Diagnostic and Statistical Manual of Mental Disorders or the International Classification of Diseases;
(iii) Comparison: studies that included a healthy control group with normal-weight individuals;
(iv) Outcomes: studies that reported the means (or medians) and standard deviations (or standard errors or interquartile ranges) for salivary cortisol and/or alpha-amylase levels;
(v) Study design: observational studies (cross-sectional, cohort, or case–control studies) or interventional trials.

The exclusion criteria were as follows:

(i) animal subjects;
(ii) review articles, meta-analysis, and conference abstracts;
(iii) unavailability of the full text article;
(iv) non-English publications.

A paper was accepted or rejected independently by two authors (TS and YN); disagreements, if any, regarding the inclusion of articles were resolved by mutual discussion among the authors.

### 2.3. Data Extraction

Two independent reviewers (TS and YN) screened the titles and abstracts for eligibility. Full-text articles were retrieved for studies that met the inclusion criteria or for articles whose eligibility could not be determined based on the abstract. A standardized data extraction form was used to collect information on study characteristics, including name of the first author, publication year, sample size, age range, body mass index (BMI), timing of saliva collection, method used for measuring cortisol and alpha-amylase, and outcome measures, such as means, medians, standard deviations (SD), standard errors (SE), and interquartile ranges (IQRs) of salivary cortisol and alpha-amylase levels. For interventional studies, baseline values that were recorded before the intervention were extracted. If the necessary data for the meta-analysis could not be collected, the corresponding author of the study was contacted. If no response was received and the data were only available in graphical format, values were extracted using WebPlotDigitizer (version 5.2) (Drevon et al., 2017).

### 2.4. Study Quality Assessment

The quality of the included studies was assessed using the Newcastle–Ottawa Scale (NOS), which was adapted for cross-sectional studies (Dalton et al., 2018). This tool evaluates three domains: selection of study groups, comparability between groups, and ascertainment of outcomes. Each study was scored on a scale from 0 to 11, with higher scores indicating better methodological quality. Studies scoring 10 or above were classified as very good, those with scores of 8–9 as good, 6–7 as satisfactory, and below 5 as unsatisfactory (Supplementary Table S3). Quality assessments were conducted independently by the two reviewers, and discrepancies, if any, were resolved through consensus.

### 2.5. Statistical Analysis

Meta-analyses were conducted using StataNow/MP 19.5 (StataCorp, College Station, TX, USA). If SEs were reported, they were converted to SDs using the following formula:

SD = SE × √n

Furthermore, when SDs were not reported but the IQRs were available, the SDs were estimated using the formula:

SD = (third quartile − first quartile)/1.3489

The standardized mean difference (SMD) was used to assess effect sizes, which were calculated using Cohen’s d. For all meta-analyses, a random-effects model was specified using the restricted maximum likelihood (REML) method. Heterogeneity across studies was assessed using the Cochran’s *Q* statistic (with corresponding *p* value) and *I*^2^ statistic. A significant *Q* statistic indicates the presence of heterogeneity among studies. An *I^2^* value of <25% was interpreted as indicating low heterogeneity, 25–75% as moderate heterogeneity, and >75% as high heterogeneity. Meta-regression analysis was conducted to examine the statistical associations between the SMD and the patients’ BMI and age. Funnel plots were created to assess the publication bias of the accepted articles. The statistical assessment of publication bias was performed using Egger’s regression test and Begg’s rank correlation test.

## 3. Results

### 3.1. Study Flow

**Figure 1:**
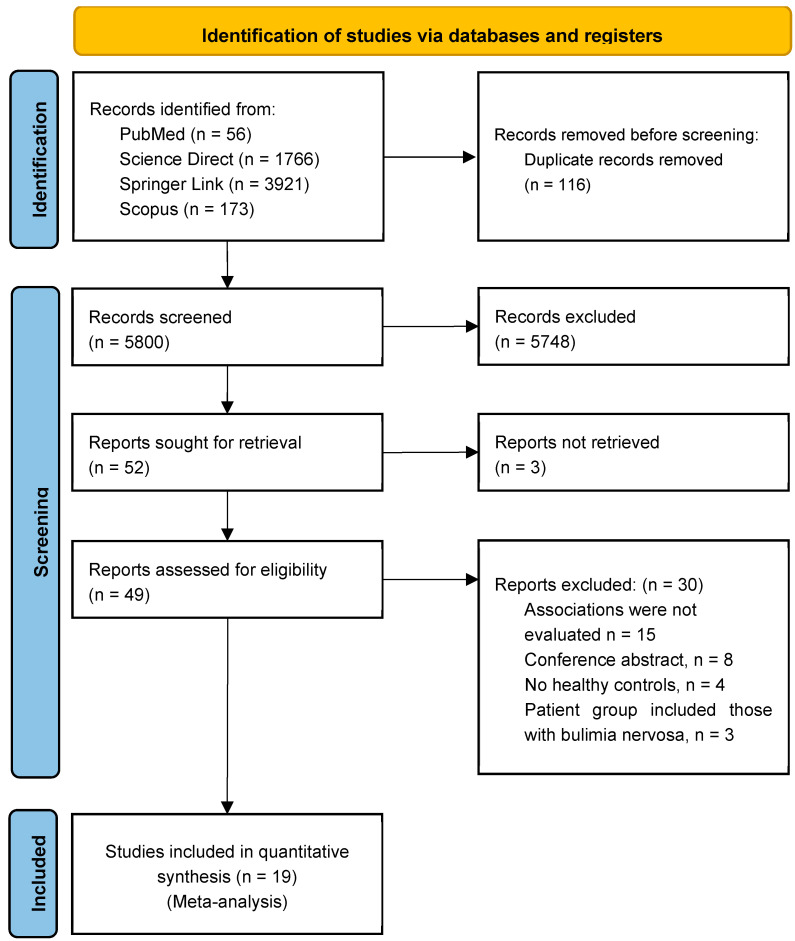
Preferred Reporting Items for Systematic Reviews and Meta-Analysis (PRISMA) flowchart.

A comprehensive database search yielded 56 articles from PubMed, 1766 from ScienceDirect, 3921 from SpringerLink, and 173 from Scopus, resulting in a total of 5916 records. After screening titles and abstracts for relevance to the objectives of this study, 49 articles were selected. Following a thorough assessment of the full texts, 30 articles that did not meet the eligibility criteria were excluded. Consequently, 19 studies were included in the meta-analysis (Figure 1).

### 3.2. Study Characteristics

Table 1 presents a summary of the main characteristics of the studies that were included in the meta-analysis. Eighteen studies included only female participants, whereas only one study included both male and female participants. These studies were published between 2001 and 2021. The study sample size ranged from 15 to 167, with a total of 997 participants (441 patients with AN and 556 healthy controls), aged between 14 and 31 years. Salivary alpha-amylase was analyzed in 2 studies, cortisol in 13 studies, and both of these biomarkers were assessed in 4 studies. In most studies, salivary biomarkers were measured using immunoassay techniques: enzyme-linked immunosorbent assay (ELISA), = 10; radioimmunoassay (RIA), *n* = 5; enzyme immunoassay (EIA), *n* = 1; and line immunoassay (LIA), *n* = 1. Only two studies employed colorimetric assays. Using the NOS adapted for cross-sectional studies, the quality assessment scores for the articles were determined as follows: 4 articles were satisfactory, 12 articles were good, and 3 were very good, with a mean score of 8.3 (Supplementary Table S4).

### 3.3. Meta-Analysis

**Figure 2:**
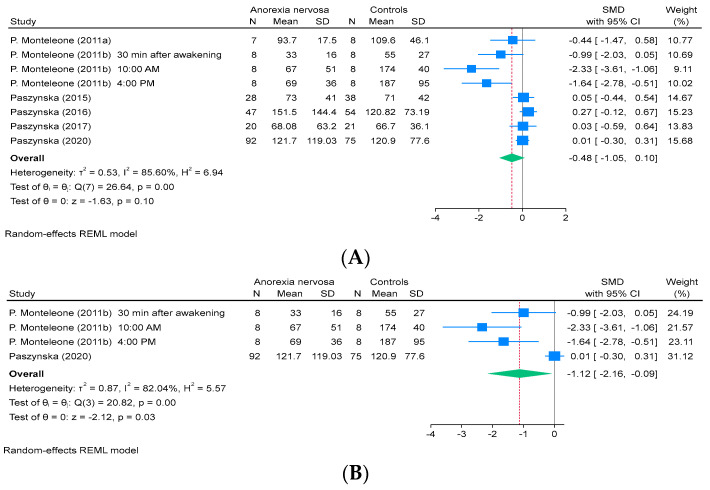
Forest plot of the (**A**) SMD for the studies that were included in the analysis of the salivary alpha-amylase levels between patients with AN and healthy controls and (**B**) sensitivity analysis excluding four studies with potentially overlapping datasets. REML, restricted maximum likelihood; SD, standard deviation; SMD, standardized mean difference; CI, confidence interval (P. Monteleone et al., 2011a, 2011b; Paszynska et al., 2015, 2016, 2017, 2020).

Salivary alpha-amylase levels were measured in 6 studies, which comprised 8 reports and included 218 patients with AN and 220 healthy controls. The meta-analysis revealed no significant difference in salivary alpha-amylase levels between patients with AN and healthy controls (SMD, −0.48; 95% CI, −1.05 to 0.10; *I*^2^, 86%; Figure 2A). However, some of the included studies were conducted by the same research groups, which raises the possibility that overlapping datasets may have been used. Therefore, we conducted a sensitivity analysis excluding four studies (P. Monteleone et al., 2011a; Paszynska et al., 2015, 2016, 2017) that may have included same dataset (*k* = 4). The results showed that salivary alpha-amylase levels were significantly lower in patients with AN than in healthy controls (SMD, −1.12; 95% CI, −2.16 to −0.09; *I*^2^, 82%; Figure 2B).

**Figure 3:**
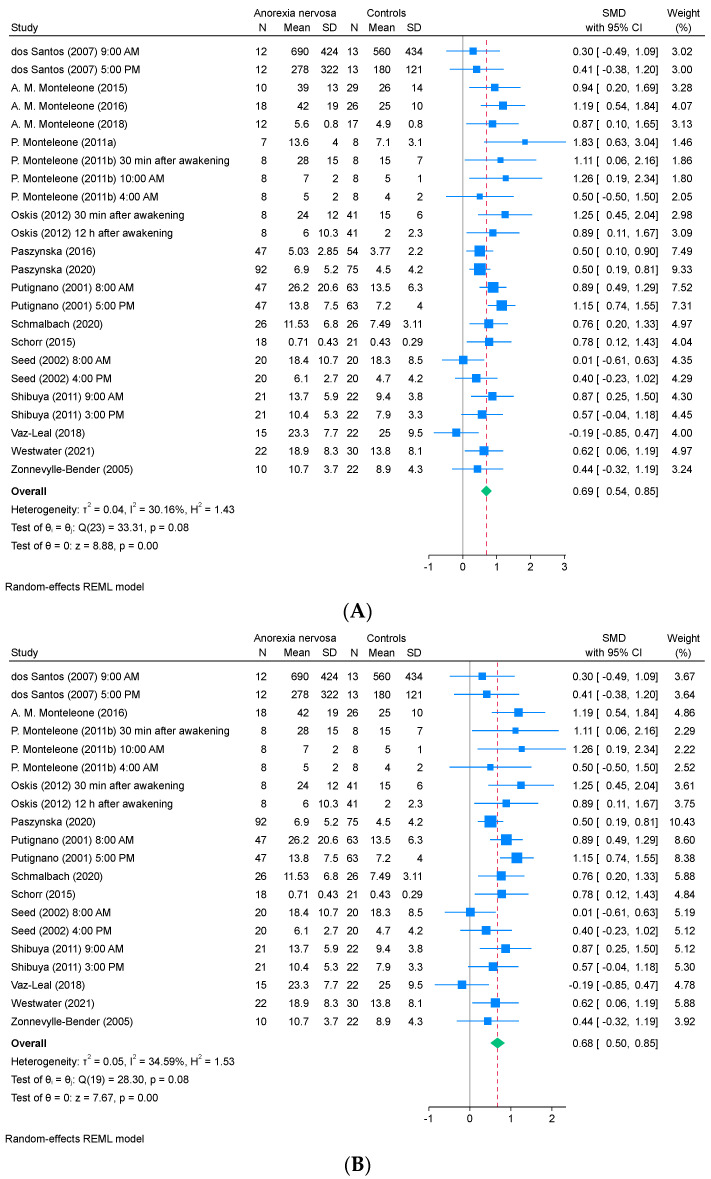
Forest plot of the (**A**) SMD for the studies that were included in the analysis of the difference in the salivary cortisol levels between patients with AN and healthy controls and (**B**) sensitivity analysis excluding four studies with potentially overlapping datasets. REML, restricted maximum likelihood; SD, standard deviation; SMD, standardized mean difference; CI, confidence interval (dos Santos et al., 2007; A. M. Monteleone et al., 2015, 2016, 2018; P. Monteleone et al., 2011a, 2011b; Oskis et al., 2012; Paszynska et al., 2016, 2020; Putignano et al., 2001; Schmalbach et al., 2020; Schorr et al., 2015; Seed et al., 2002; Shibuya et al., 2011; Vaz-Leal et al., 2018; Westwater et al., 2021; Zonnevylle-Bender et al., 2005).

Salivary cortisol levels were assessed in 17 studies, which comprised 24 reports and involved 517 patients with AN and 672 healthy controls. The results indicated that salivary cortisol levels were significantly higher in patients with AN than in healthy controls (SMD, 0.69; 95% CI, 0.54 to 0.85; *I*^2^, 30%; Figure 3A). A sensitivity analysis excluding 4 studies (P. Monteleone et al., 2011a; A. M. Monteleone et al., 2015, 2018; Paszynska et al., 2016) showed that the overall pattern of results remained largely unchanged (SMD, 0.68; 95% CI, 0.50 to 0.85; *I*^2^, 35%; Figure 3B).

### 3.4. Subgroup Analysis

**Figure 4:**
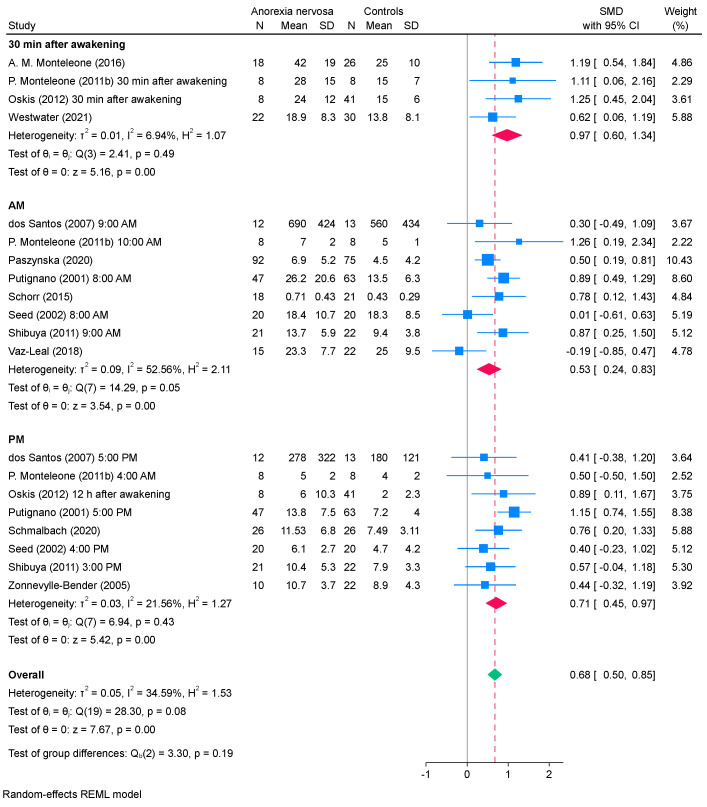
Results of subgroup analysis for the salivary cortisol levels between patients with AN and healthy controls based on the timing of saliva collection. REML, restricted maximum likelihood; SD, standard deviation; SMD, standardized mean difference; CI, confidence interval (dos Santos et al., 2007; A. M. Monteleone et al., 2016; P. Monteleone et al., 2011b; Oskis et al., 2012; Paszynska et al., 2020; Putignano et al., 2001; Schmalbach et al., 2020; Schorr et al., 2015; Seed et al., 2002; Shibuya et al., 2011; Vaz-Leal et al., 2018; Westwater et al., 2021; Zonnevylle-Bender et al., 2005).

**Figure 5:**
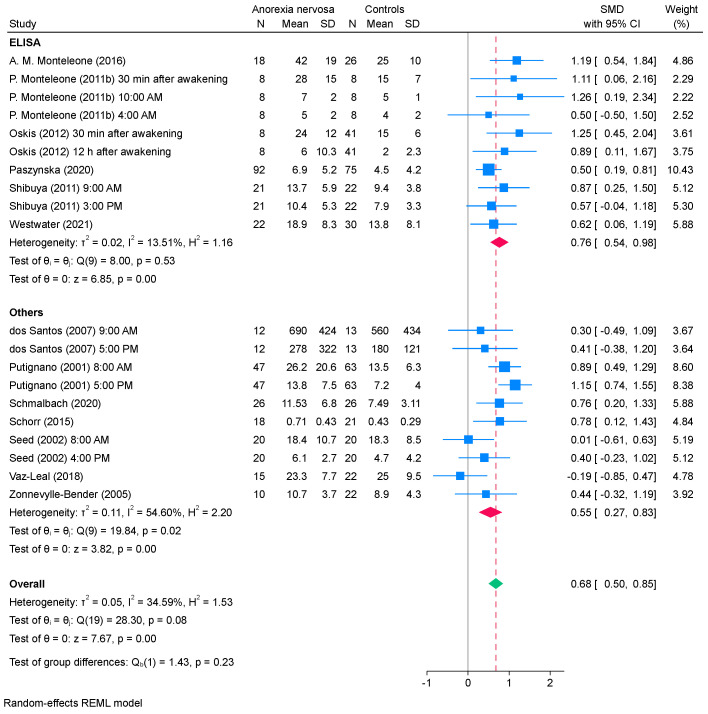
Results of subgroup analysis for the salivary cortisol levels between patients with AN and healthy controls based on the measurement method. REML, restricted maximum likelihood; SD, standard deviation; SMD, standardized mean difference; CI, confidence interval (dos Santos et al., 2007; A. M. Monteleone et al., 2016; P. Monteleone et al., 2011b; Oskis et al., 2012; Paszynska et al., 2020; Putignano et al., 2001; Schmalbach et al., 2020; Schorr et al., 2015; Seed et al., 2002; Shibuya et al., 2011; Vaz-Leal et al., 2018; Westwater et al., 2021; Zonnevylle-Bender et al., 2005).

Figure 4 and Figure 5 present the results of the subgroup analysis conducted after excluding four studies (*k* = 20) assessing salivary cortisol. The subgroup analysis based on the timing of saliva collection showed that, in studies where saliva was collected within the first 30 min after awakening, salivary cortisol levels were significantly higher in patients with AN than those in healthy controls, with low heterogeneity detected (SMD, 0.97; 95% CI, 0.60 to 1.34; *I*^2^, 7%). In the subgroup analysis based on the measurement method, studies using ELISA showed significantly higher salivary cortisol levels in patients with AN than those in healthy controls (SMD, 0.76; 95% CI, 0.54 to 0.98), with low heterogeneity (*I*^2^ = 14%). Similarly, studies employing other methods found significantly elevated salivary cortisol levels in patients with AN compared with healthy controls (SMD, 0.55; 95% CI, 0.27 to 0.83). However, a moderate heterogeneity was observed (*I*^2^ = 55%).

### 3.5. Meta-Regression Analysis

Table 2 presents the results of the meta-regression analysis (*k* = 20). In a simple meta-regression analysis, neither the age nor BMI of patients significantly affected SMD for salivary cortisol.

### 3.6. Publication Bias

**Figure 6:**
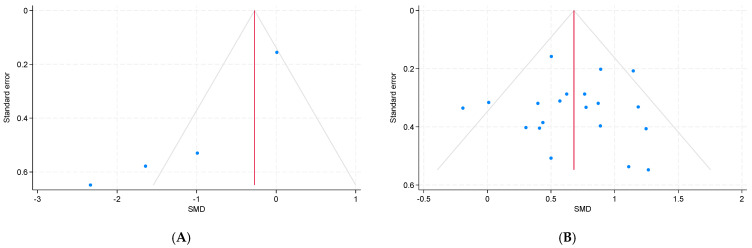
Funnel plot of the results of the random-effect model for studies that were included in the analyses of salivary (**A**) alpha-amylase and (**B**) cortisol levels.

Figure 6 presents a funnel plot. For the studies assessing alpha-amylase (*k* = 4; Figure 6A), Egger’s test indicated significant publication bias (*p* < 0.001), whereas Begg’s test did not yield significant results (*p* = 0.09). In contrast, for the studies assessing cortisol (*k* = 20; Figure 6B), neither Egger’s test nor Begg’s test showed evidence of publication bias (*p* = 0.96, *p* = 0.82, respectively).

## 4. Discussion

There is a growing body of evidence, which shows that salivary alpha-amylase and cortisol levels are associated with symptoms of AN. In this systematic review and meta-analysis, we aimed to compare these levels between patients with AN and healthy controls. In the primary analysis, no significant difference was observed in salivary alpha-amylase levels between patients with AN and healthy controls. However, a sensitivity analysis excluding studies with potentially overlapping samples revealed a significant difference between the two groups. In contrast, salivary cortisol levels were significantly elevated in patients with AN. Significantly elevated cortisol levels in patients with AN reflect chronic hyperactivation of the HPA axis. Persistent elevations in cortisol can disrupt the regulation of appetite-related hormones, such as ghrelin and peptide YY, thereby contributing to enhanced postprandial satiety and suppression of food intake, ultimately promoting the maintenance of low body weight (Mancuso et al., 2020). Additionally, elevated cortisol levels are associated with increased cognitive rigidity and impaired flexibility in eating-related behaviors (Roberts et al., 2010; Abbate-Daga et al., 2011). These characteristics are frequently observed in patients with AN and represent plausible mechanistic pathways underlying the persistence of restrictive eating patterns.

Subgroup analysis indicated that the timing of saliva collection influenced the observed mean difference in cortisol levels between patients with AN and healthy controls. Specifically, studies in which saliva was collected immediately after waking exhibited a large SMD and low heterogeneity between studies. These results align with the well-established diurnal pattern of cortisol, which peaks immediately after awakening and declines steadily throughout the day (Weitzman et al., 1971; Pruessner et al., 1997; Adam & Kumari, 2009). This pattern is regulated by the suprachiasmatic nucleus, the brain’s central circadian pacemaker, and can be disrupted by external factors, such as sleep disturbances, dietary habits, and stress (Larsen et al., 1994; Easton et al., 2004; Zhai et al., 2022). Owing to these biological characteristics, it is widely recommended, in both research and clinical fields, to collect salivary cortisol samples immediately upon awakening, 30 min after awakening, or at least during the morning hours. Our findings support this recommendation and highlight the importance of standardizing collection times to improve diagnostic reliability and facilitate cross-study comparability.

In addition, no differences in SMD were observed between ELISA and other analytical methods for measuring salivary cortisol. Although few studies have directly compared salivary cortisol levels across different analytical techniques, an unpublished report indicates a strong correlation between measurements obtained via ELISA and liquid chromatography–tandem mass spectrometry (LC-MS/MS) (Lee et al., 2023). However, LC-MS/MS demonstrated superior accuracy, particularly in the low concentration range. Another study that compared salivary cortisol values obtained through RIA and LC-MS/MS in the context of a diagnosis of Cushing’s syndrome found that although discrepancies between two methods were minimal in healthy individuals, RIA exhibited a higher false-positive rate, whereas LC-MS/MS showed superior specificity and diagnostic accuracy (Baid et al., 2007). Overall, LC-MS/MS is considered the most accurate method for measuring salivary biomarkers and is especially recommended for detecting low concentrations or when diagnostic precision is critical. In contrast, ELISA and RIA are simpler and more cost-effective methods but are prone to overestimating concentrations and may increase the risk of misdiagnosis, especially near diagnostic thresholds. Although none of the studies included in this meta-analysis employed LC-MS/MS, no substantial differences in cortisol levels were found when using other common methods, such as ELISA, RIA, or EIA.

In recent years, research examining the relationship between salivary cortisol and BMI has advanced. However, findings remain inconsistent, and both the direction and strength of the association appear to vary depending on the specific disorder being studied. For example, a previous study reported a negative correlation between BMI and salivary cortisol levels in patients with functional hypothalamic amenorrhea (Koukoubanis et al., 2023). Conversely, in women with obesity and binge eating disorder, the severity of binge eating behavior plays a more substantial role in modulating salivary cortisol secretion than does the BMI (Coutinho et al., 2007). In addition to the direct relationship between BMI and salivary cortisol levels, emerging evidence suggests that patient characteristics, such as age (Kiess et al., 1995; Ahn et al., 2007; Ceccato et al., 2015) and sex (Paris et al., 2010; Hinkelmann et al., 2012), may influence the circadian rhythm of salivary cortisol secretion. However, the results of the present meta-regression analysis revealed no significant associations between differences in salivary cortisol levels and either BMI or age. These findings suggest that BMI and age are unlikely to be major confounding factors of salivary cortisol levels in patients with AN. One possible explanation for this finding is that the chronic physiological adaptations associated with AN, such as altered HPA axis functioning, may override or obscure the effects of age and body composition. Additionally, the methodological heterogeneity across studies, such as the timing of sample collection, may have limited our ability to detect meaningful associations. Future research should employ standardized methodologies to clarify these relationships.

In the case of salivary alpha-amylase, the sensitivity analysis produced discrepant results, with a significant difference emerging after studies with potentially overlapping data were excluded. This finding suggests that the primary analysis may have been obscured by overlapping datasets or non-independence among samples. Moreover, similar to cortisol, salivary alpha-amylase is widely used as a noninvasive biomarker of physiological stress; however, these two biomarkers differ markedly in their physiological responses: salivary cortisol reflects HPA axis activity and is associated with chronic stress responses (Hellhammer et al., 2009), whereas salivary alpha-amylase reflects SAM system activity and responds rapidly to acute stress (Strahler et al., 2010). Given these differences, salivary alpha-amylase may not be a useful marker for diagnosing patients with AN. However, few studies have examined the relationship between patients with AN and salivary alpha-amylase; therefore, further research is needed.

This research has some limitations that should be acknowledged. First, this review was limited to a small number of databases and did not include major indexing services such as MEDLINE and Web of Science. As a result, some relevant studies may have been missed, and the overall coverage of the literature may not be as comprehensive as intended. Future reviews should incorporate additional databases to ensure a more exhaustive and representative search of the existing literature. Second, the number of studies included in the meta-analysis was relatively small. As the systematic literature search yielded only a limited number of eligible studies, the reliability and generalizability of the findings may be constrained. In particular, the meta-analysis of salivary alpha-amylase included too few studies to permit subgroup analyses. To enhance the robustness of future meta-analytic assessments, a larger body of primary research is necessary. Third, clinical symptomatology in patients with AN may contribute to the variability observed in biomarker levels. Relevant clinical factors include duration of illness, medication use, and the presence of mental illness (e.g., depression, anxiety disorders). However, these variables were often insufficiently reported, limiting the feasibility of conducting more detailed subgroup or meta-regression analyses and likely contributing to clinical heterogeneity. Fourth, the causal relationship was unclear. As many of the studies included in the meta-analysis were cross-sectional, it is unclear whether eating disorders increase cortisol levels or whether high cortisol levels cause eating disorders. Therefore, longitudinal studies will be necessary in future research.

## 5. Conclusions

This meta-analysis showed that salivary cortisol levels were significantly elevated in patients with AN. This result underscores the potential utility of salivary cortisol as a noninvasive biomarker for AN and suggests its application for early detection or clinical monitoring of patients with AN. Future research should include longitudinal designs to determine the direction of the causal relationship between salivary biomarkers and AN. Additionally, standardizing clinical variables such as duration of illness, comorbidities, and medication use is essential for enhancing comparability across studies.

## Figures and Tables

**Table 1:** Characteristics of the included studies.

No.	Author, Publication Year	Gender	Sample	N	Age	BMI	Duration of Illness	Biomarker of Interest	Measurement Method	Collection Time	Quality
1	dos Santos et al. (2007)	Female	AN HC	12 13	19.5 (median) 21.0 (median)	15.6 (median) 21.4 (median)	NR	Cortisol	RIA	9:00 AM, 5:00 PM, and 11:00 PM	8
2	A. M. Monteleone et al. (2015)	Female	Non-maltreated women with AN HC	10 29	27.3 ± 10.0 27.6 ± 6.7	17.0 ± 1.3 21.5 ± 2.8	NR	Cortisol	ELISA	Awakening and 15, 30, and 60 min after awakening	8
3	A. M. Monteleone et al. (2016)	Female	Underweight women with AN HC	18 26	26.9 ± 5.6 26.3 ± 5.6	16.1 ± 1.7 20.9 ± 2.7	NR	Cortisol	ELISA	Awakening and 15, 30, and 60 min after awakening	8
4	A. M. Monteleone et al. (2018)	Female	Non-maltreated woman with AN HC	12 17	23.33 ± 5.2 26.00 ± 2.5	16.88 ± 1.5 21.86 ± 2.5	4.67 ± 3.47 years	Cortisol	ELISA	Between 2:30 PM and 4:30 PM	8
5	P. Monteleone et al. (2011a)	Female	AN HC	7 8	20.2 ± 2.2 23.6 ± 2.2	16.3 ± 1.2 21.1 ± 2.4	5.8 ± 4.2 year	Alpha-amylase, Cortisol	ELISA	Between 3:30 PM and 5:00 PM	7
6	P. Monteleone et al. (2011b)	Female	AN HC	8 8	21.6 ± 3.6 25.5 ± 4.5	17.2 ± 1.5 20.5 ± 1.9	NR	Alpha-amylase, Cortisol	ELISA	Awakening and 15, 30, and 60 min after awakening, 10:00 AM, 12:00 PM, 4:00 PM, 6:00 PM, 7:00 PM, and 8:00 PM	8
7	Oskis et al. (2012)	Female	AN HC	8 41	15.13 ± 1.64 15.46 ± 1.53	18.56 ± 1.09 22.05 ± 3.54	NR	Cortisol	ELISA	Awakening and 30 min, and 12 h after awakening	7
8	Paszynska et al. (2015)	Female	AN HC	28 38	15 ± 2 14 ± 1	NR	1.2 ± 0.6 years	Alpha-amylase	Colorimetric assay	Between 9:00 AM and 12:00 PM	9
9	Paszynska et al. (2016)	Female	AN HC	47 54	15 ± 2 15 ± 2	14.18 ± 1.31 19.96 ± 1.33	11 ± 5 months	Alpha-amylase, Cortisol	ELISA	Between 9:00 AM and 11:00 AM	9
10	Paszynska et al. (2017)	Female	AN HC	20 21	15 ± 2 16 ± 2	14.09 ± 1.3 20.00 ± 1.5	14 ± 9 months	Alpha-amylase	Colorimetric assay	Between 9:00 AM and 12:00 PM	8
11	Paszynska et al. (2020)	Female	AN HC	92 75	15.02 ± 2.4 15.4 ± 2.3	14.3 ± 1.4 19.8 ± 1.7	10.5 ± 6 months	Alpha-amylase, Cortisol	ELISA	Between 9:00 AM and 10:00 AM	10
12	Putignano et al. (2001)	Female	Untreated AN HC	47 63	23.0 ± 6.3 31.2 ± 10.2	13.7 ± 2.26 20.8 ± 2.14	NR	Cortisol	RIA	8:00 AM, 5:00 PM, and 12:00 AM	8
13	Schmalbach et al. (2020)	Both	AN HC	26 26	26.5 ± 6.1 25.0 ± 5.3	19.3 ± 3.4 23.08 ± 3.3	NR	Cortisol	LIA	Between 2:00 PM and 4:00 PM	10
14	Schorr et al. (2015)	Female	AN HC	18 21	26 ± 6 27 ± 7	18.2 ± 1.0 22.3 ± 1.4	NR	Cortisol	EIA	7:00 AM	7
15	Seed et al. (2002)	Female	AN HC	20 20	29.1 ± 8.4 29.3 ± 8.6	14.2 ± 2.1 22.4 ± 3.1	NR	Cortisol	RIA	8:00 AM, 12:00 PM, 4:00 PM, and 8:00 PM	8
16	Shibuya et al. (2011)	Female	AN before inpatient treatment HC	21 22	14.4 ± 1.4 14.0 ± 1.2	13.0 ± 1.5 19.3 ± 2.1	11.0 ± 9.7 months	Cortisol	ELISA	9:00 AM, 11:00 AM, 1:00 PM, 3:00 PM, 5:00 PM, and 7:00 PM	8
17	Vaz-Leal et al. (2018)	Female	AN HC	15 22	22.0 ± 3.6 21.7 ± 2.3	16.7 ± 0.8 21.6 ± 1.1	4.9 ± 2.5 years	Cortisol	RIA	8:00 AM	8
18	Westwater et al. (2021)	Female	AN HC	22 30	24.6 ± 4.7 23.9 ± 3.5	16.4 ± 1.4 21.9 ± 2.1	9.0 ± 5.8 years	Cortisol	ELISA	Awakening and 30, 45, and 60 min after awakening	11
19	Zonnevylle-Bender et al. (2005)	Female	AN HC	10 22	15.5 ± 1.8 14.9 ± 1.1	16.2 ± 1.2 NR	7–15 months	Cortisol	RIA	Between 12:00 PM and 6:00 PM	7

AN, anorexia nervosa; HC, healthy controls; BMI, body mass index; NR, not reported; RIA, radioimmunoassay; ELISA, enzyme-linked immuno-sorbent assay; LIA, line immunoassay; EIA, enzyme immunoassay.

**Table 2:** Results of simple meta-regression analysis.

Covariate	Reports	Coefficient	95% CI	*p*
Age	20	−0.005	−0.41, 0.30	0.77
BMI	20	0.021	−0.07, 1.50	0.11

BMI, body mass index; CI, confidence interval.

## Data Availability

The data relevant to this study can be found within the article and its Supplementary Files.

## Supplementary Materials

The following supporting information can be downloaded at: https://www.mdpi.com/article/10.3390/ejihpe15120260/s1, Table S1: Detailed search strategy; Table S2: The PRISMA Checklist; Table S3: Newcastle-Ottawa Quality Assessment Scale adapted for cross-sectional studies; Table S4: Quality assessment for each study.

## Abbreviations

The following abbreviations are used in this manuscript:

AN	anorexia nervosa
BMI	body mass index
BN	bulimia nervosa
CI	confidence interval
ELISA	enzyme-linked immunosorbent assay
EIA	enzyme immunoassay
HPA	hypothalamic–pituitary–adrenal
IQR	interquartile range
LC-MS/MS	liquid chromatography–tandem mass spectrometry
LIA	line immunoassay
NOS	Newcastle–Ottawa Scale
PRISMA	Preferred Reporting Items for Systematic Reviews and Meta-Analyses
REML	restricted maximum likelihood
RIA	radioimmunoassay
SAM	sympatho-adrenomedullary
SD	standard deviation
SE	standard error
SMD	standardized Mean Difference
