# Supplementary material for: Evaluating Salivary Cortisol and Alpha-Amylase as Candidate Biomarkers in Anorexia Nervosa: A Systematic Review and Meta-Analysis
Source: Eur J Investig Health Psychol Educ. 2025 Dec 17;15(12):260. doi: 10.3390/ejihpe15120260 (PMC12731984; doi:10.3390/ejihpe15120260)
Supplement: Supplementary file 1 [file ejihpe-15-00260-s001.zip › Table S1.pdf]

Table S1 Detailed search strategy

Database	Search terms
PubMed	("Feeding and Eating Disorders"[MeSH] OR "Feeding and Eating Disorder*" OR "Anorexia"[MeSH] OR "Anorexia" OR "Anorexia Nervosa"[MeSH] OR "Anorexia nervosa" OR "Binge-Eating Disorder"[MeSH] OR "Binge-Eating Disorder*") AND ("Saliva"[MeSH] OR "Sariva*") AND ("Biomarker*" OR "Hydrocortisone"[MeSH] "Hydrocortisone*" OR "Amylases"[MeSH] OR "Amylase*" OR "alpha-Amylases"[MeSH] OR "alpha-Amylase*")
ScienceDirect	("Eating Disorders" OR "Anorexia Nervosa" OR "Binge-Eating Disorder") AND ("Saliva" OR "Salivary") AND ("Biomarkers" OR "Cortisol" OR "Amylases" OR "alpha-Amylases")
Scopus	TITLE-ABS-KEY("Eating Disorders" OR "Anorexia Nervosa" OR "Anorexia" OR "Binge-Eating Disorder") AND TITLE-ABS-KEY("Saliva" OR "Salivary") AND TITLE-ABS-KEY("Biomarkers" OR "Cortisol" OR "Amylases" OR "alpha-Amylases")
SpringerLink	("Eating Disorders" OR "Anorexia Nervosa" OR "Anorexia" OR "Binge-Eating Disorder") AND ("Saliva" OR "Salivary") AND ("Biomarkers" OR "Cortisol" OR "Amylases" OR "alpha-Amylases")
