# Supplementary material for: Evaluating Salivary Cortisol and Alpha-Amylase as Candidate Biomarkers in Anorexia Nervosa: A Systematic Review and Meta-Analysis
Source: Eur J Investig Health Psychol Educ. 2025 Dec 17;15(12):260. doi: 10.3390/ejihpe15120260 (PMC12731984; doi:10.3390/ejihpe15120260)
Supplement: Supplementary file 1 [file ejihpe-15-00260-s001.zip › Table S3.pdf]

Table S3 Newcastle-Ottawa Quality Assessment Scale adapted for cross-sectional studies.

Selection: (Maximum 5 stars)

1) Representativeness of the sample:
   a) Truly representative of the average in the target population. ** (all subjects or random sampling)
   b) Somewhat representative of the average in the target population. * (non-random sampling)
   c) Selected group of users.
   d) No description of the sampling strategy.

2) Sample size:
   a) Justified and satisfactory ($n \geq 50$). *
   b) Not justified ($n < 50$).

3) Ascertainment of the exposure:
   a) Validated measurement tool. **
   b) Non-validated measurement tool, but the tool is available or described. *
   c) No description of the measurement tool.

Comparability: (Maximum 3 stars)

1) The subjects in different outcome groups are comparable, based on the study design or analysis. Confounding factors are controlled.
   a) The study controls for the most important factor (gender). *
   b) The study control for an additional important factor (age). *
   c) The study control for an additional important factor (smoking). *

Outcome: (Maximum 3 stars)

1) Assessment of the outcome:
   a) Validated measurement method. **
   b) Non-validated measurement method, but the method is available or described. *
   c) No description of the measurement tool.

2) Statistical test:
   a) The statistical test used to analyze the data is clearly described and appropriate, and the measurement of the association is presented, including confidence intervals and the probability level (p value). *
   b) The statistical test is not appropriate, not described or incomplete.

Studies with scores of 0-5, 6-7, 8-9, and 10-11 were considered as Unsatisfactory Studies, Satisfactory Studies, Good Studies, and Very Good Studies, respectively.
