# Supplementary material for: Evaluating Salivary Cortisol and Alpha-Amylase as Candidate Biomarkers in Anorexia Nervosa: A Systematic Review and Meta-Analysis
Source: Eur J Investig Health Psychol Educ. 2025 Dec 17;15(12):260. doi: 10.3390/ejihpe15120260 (PMC12731984; doi:10.3390/ejihpe15120260)
Supplement: Supplementary file 1 [file ejihpe-15-00260-s001.zip › Table S4.pdf]

Table S4 Quality assessment for each study.

Studies	**Selection**: (Maximum 5 stars)			**Comparability:** (Maximum 3 stars)			**Outcome:** (Maximum 3 stars)		**Total score**
	Representativeness of the sample	Sample size	Ascertainment of the exposure	Most important factor (gender)	Additional important factor (age)	Additional important factor (smoking)	Assessment of the outcome	Statistical test	
dos Santos et al., 2007	*		**	*	*		**	*	8
A. M. Monteleone et al., 2015	*		**	*	*		**	*	8
A. M. Monteleone et al., 2016	*		**	*	*		**	*	8
A. M. Monteleone et al., 2018	*		**	*	*		**	*	8
P. Monteleone et al., 2011a	*		**	*			**	*	7
P. Monteleone et al., 2011b	*		**	*	*		**	*	8
Oskis et al., 2012	*			*	*	*	**	*	7
Paszynska et al., 2015		*	**	*	*	*	**	*	9
Paszynska et al., 2016		*	**	*	*	*	**	*	9
Paszynska et al., 2017			**	*	*	*	**	*	8
Paszynska et al., 2020	*	*	**	*	*	*	**	*	10
Putignano et al., 2001	*	*	**	*			**	*	8
Schmalbach et al., 2020	**	*	**	*	*		**	*	10
Schorr et al., 2015			**	*	*		**	*	7
Seed et al., 2002	*		**	*	*		**	*	8
Shibuya et al., 2011	*		**	*	*		**	*	8
Vaz-Leal et al., 2018	*		**	*	*		**	*	8
Westwater et al., 2021	**	*	**	*	*	*	**	*	11
Zonnevylle-Bender et al., 2005			**	*	*		**	*	7
